# Reverse Osmosis Membrane Cleaning Optimization from Textile Dyeing Wastewater Reuse Applications

**DOI:** 10.3390/membranes16010029

**Published:** 2026-01-04

**Authors:** Zhengwei Wang, Rulu Ouyang, Guorui Zhang, Chunhai Wei, Shiming Ji, Qixuan Li, Chunyang Tao, Hongwei Rong

**Affiliations:** 1 Department of Municipal Engineering, School of Civil Engineering and Transportation, Guangzhou University, Guangzhou 510006, China; anhwzw@163.com (Z.W.); ouyangrulu2022@163.com (R.O.); 2 Guangdong Shunkong Zihua Technology Co., Ltd., Foshan 528000, China; jsmsunny@163.com (S.J.); kite035@126.com (Q.L.); 19865685978@163.com (C.T.); 3 Guangzhou Water Supply Co., Ltd., Guangzhou 510600, China; 4 Department of Civil and Environmental Engineering, Texas A&M University, College Station, TX 77843-3122, USA; guoruizhang9@gmail.com; 5 Key Laboratory for Water Quality and Conservation of the Pearl River Delta, Ministry of Education, Guangzhou 510006, China

**Keywords:** combined cleaning, conductivity, constant-pressure crossflow filtration, membrane fouling, specific flux

## Abstract

Reverse osmosis (RO) is the key process for textile dyeing wastewater reuse applications. Membrane fouling reduces both permeability and rejection capability, negatively affecting the technological economy of RO process. Membrane cleaning is critical to recovery of the permeability of fouled RO membranes. Based on multi-batch filtration and cleaning experiments, this study systematically evaluated the RO membrane fouling potential of pre-treated textile dyeing wastewater by a membrane bioreactor and the recovery performance of fouled RO membranes after different cleaning methods. A significant decline (more than 15%) in RO membrane permeability occurred after RO membrane permeate production of 625 L/m^2^ at a water recovery ratio of 60%. Protein-like substances and soluble microbial products were identified as the primary organic foulants via three-dimensional fluorescence excitation-emission matrix spectrometry (3D-FEEM). The single forward flushing with either pure water, acid, alkaline, or sodium hypochlorite solutions with a low active chlorine concentration showed very limited recovery of fouled RO membrane permeability. The combined forward flushing with acid followed by alkaline solutions restored fouled membrane permeability by up to 87% of a new RO membrane. The addition of pure water backwashing at a transmembrane pressure (TMP) of 0.5 MPa after both acid and alkaline solutions combined forward flushing restored fouled membrane permeability by up to 97% of a new RO membrane but deteriorated the rejection capability of the RO membrane. The backwashing parameters were further optimized at a TMP of 0.125 MPa and crossflow velocity (CFV) of 0.5 m/s, achieving fouled RO membrane permeability by up to 96% of a new RO membrane, and there were no negative effects on the rejection capability of the RO membrane. Alkaline forward flushing followed by pure water backwashing was the dominant contributor for fouled RO membrane permeability recovery. A preliminary economic analysis showed that the total chemical cost per RO production was 0.763 CNY/m^3^ and could be further reduced via removing acid cleaning and replacing combined alkaline flushing and pure water backwashing with alkaline backwashing.

## 1. Introduction

Due to the dense structure of the separation layer, reverse osmosis (RO) membranes can retain the vast majority of inorganic and organic substances [1,2], leading to their intensive application in the production of high-quality reclaimed water. They have been applied on a large scale in seawater desalination and advanced treatment of difficult-to-treat organic wastewater, including sea sand washing wastewater, textile dyeing wastewater, coking wastewater, and landfill leachate [3,4].

The textile industry is one of the largest wastewater producers among all industrial sectors in China, discharging wastewater of 1.84 billion tons and accounting for 10.1% of the total industrial wastewater discharge amount in 2015 [5]. In the context of textile dyeing wastewater, which is characterized by high chromaticity, substantial salinity, and complex compositions of dyes, sizing agents, and additives [6,7], RO technology presents a promising solution for achieving water reuse and meeting stringent discharge standards [8,9]. However, its application in this field is severely hampered by membrane fouling [10]. The diverse organic and inorganic constituents in dyeing wastewater can form a gel layer and scaling due to concentration polarization, leading to a significant decline in permeate flux and an increase in operating pressure. Consequently, frequent chemical cleaning is required, which shortens the membrane lifespan and elevates operational costs [8,11,12].

Many scholars are dedicated to searching for ways to reduce the fouling potential of RO membranes and finding efficient cleaning methods to extend membrane lifespan [13,14,15]. Ćurić et al. [16] achieved membrane fouling control by coupling suitable pretreatment technologies with membrane technology. Results indicate that the combined ultrafiltration (UF)+RO process outperforms sand filtration and coagulation sedimentation in removing turbidity, color, and total organic carbon (TOC), playing a significant role in reducing the fouling potential of membranes in textile dyeing wastewater. Srisukphun et al. [17] elucidated the fouling mechanism of RO membranes in textile wastewater applications. They reported that the repulsive force between the anionic charge of reactive dyes and anionic surfactants prevents their aggregation, constituting a primary factor affecting membrane flux. Zeng [18] and Ma [19] conducted a series of tests on biological removal and oxidative adsorption of dyes and surfactants in textile wastewater, aiming to achieve adsorption and degradation of scaling sources using sulfate-reducing bacteria (SRB) or oxidized graphene, respectively. Results demonstrated adsorption and degradation rates exceeding 99%, significantly reducing membrane scaling risks.

Membrane cleaning methods are primarily categorized into physical and chemical cleaning. Physical cleaning relies on mechanical force to scrape foulants from the membrane surface under varying hydraulic conditions [20]. Chemical cleaning involves chemical agents reacting with membrane foulants to alter their structure and reduce the cohesive forces between foulants and the membrane surface, thereby restoring membrane permeability [21]. The commonly used chemical agents include acids, alkalis, surfactants, and chelating agents. Acids, such as citric acid, hydrochloric acid, nitric acid, and sulfuric acid, are used to remove inorganic foulants [22]. Alkalis, mainly sodium hydroxide, are used to remove organic and biological foulants [23]. In practical applications, intensive chemical cleaning is indispensable during RO treatment of textile wastewater [24]. Membrane aging necessitates refined cleaning protocols that ensure thorough cleaning while minimizing chemical corrosion of the membrane itself [14]. However, there is little information on the RO membrane cleaning performance of different cleaning methods (especially backwashing) for textile dyeing wastewater reuse applications in the literature.

In this study, the fouling potential of a typical RO membrane by biologically treated textile dyeing wastewater was firstly evaluated. Then, the RO membrane cleaning performance of pure water, acid, and alkaline solutions, and their combined forward flushing methods, was tested. Finally, an effective cleaning protocol for alkaline forward flushing followed by optimized pure water backwashing was proposed to recover RO membrane permeability without reducing RO membrane rejection capability. This study could provide a valuable reference for RO membrane cleaning for textile dyeing wastewater reuse applications.

## 2. Materials and Methods

### 2.1. Laboratory-Scale RO Setup and RO Membranes

**Figure 1:**
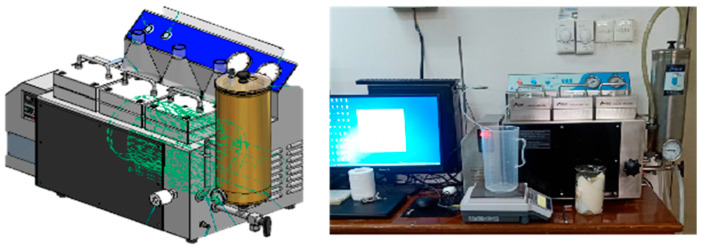
Constant-pressure crossflow RO setup with concept diagram (**left**) and field photo (**right**).

A constant-pressure crossflow RO setup (Figure 1, FlowMem0021-MP, Xiamen Filter & Membrane Technology Co., Ltd., Xiamen, China) was used for multi-batch filtration and cleaning experiments in this study and its detailed description was shown in previous studies [24]. The RO membrane used in this study is a flat-sheet membrane from Vontron Technology Co., Ltd. (Guiyang, China), model HOR (referring to high oxidation resistance), with the type being a polyamide composite membrane. Prior to testing, membrane discs of appropriate size must be cut using a template, resulting in the RO membrane sheet having a rectangular dimension of 16 cm × 6 cm with filtration area of 96 cm^2^. New membranes should then be placed in pure water for 30 min to remove glycerol from the surface.

### 2.2. Wastewater Sample and Examination Methods

Effluent samples were collected from a pilot-scale membrane bioreactor (MBR) treating textile dyeing and finishing wastewater (TDFW) in Foshan, Guangdong Province, China, and used as RO feedwater (Table 1). The effluent appeared clear and yellowish-brown, with no suspended solids (SSs) observed, which can be attributed to the use of an ultrafiltration membrane with a mean pore size of 40 nm. The water quality parameters of the RO feed and RO permeate standards for textile water reuse in China (FZ/T 01107-2011) [25] are summarized in Table 1. RO permeate in this study always satisfied this standard.

As reported in previous studies [24], water quality was measured according to the standard methods [26]. pH and conductivity were measured using a pH and conductivity dual-function meter (HQ4300, Hach, Loveland, CO, USA). Chemical oxygen demand (COD) was determined by potassium dichromate oxidation followed by titration with ferrous ammonium sulfate. Total organic carbon (TOC) was analyzed using an organic carbon analyzer (TOC-L, Shimadzu, Kyoto, Japan). Suspended solids (SS) were quantified by the gravimetric method after heating. Chroma was assessed using the dilution visual colorimetric method. The concentrations of Fe, Mn, and total hardness (as CaCO_3_) were measured with an inductively coupled plasma spectrometer (iCAP 7000, Thermo Scientific, Waltham, MA, USA). Dissolved organic matter (DOM) in RO feed and permeate was characterized using three-dimensional fluorescence excitation-emission matrix spectrometry (3D-FEEM) [27].

### 2.3. Batch RO Tests and Analytical Methods

The evaluation of RO membrane fouling potential in TDFW reuse and its optimal cleaning program were divided into three main parts:

(1) RO Membrane Fouling Potential in TDFW Reuse. This stage focused on multi-batch filtration at an operating pressure (TMP) of 2 MPa, crossflow velocity (CFV) of 1.0 m/s, temperature of 20 °C, and each batch running until 60% water recovery. The aim was to identify the filtration volume per unit membrane area or filtration batches at which significant RO membrane fouling occurred (i.e., relative permeability decrease by 15% prior to membrane cleaning according to the general practice for RO industry) [28,29,30], thereby establishing an experimental basis for subsequent membrane cleaning studies. The specific flux (i.e., the linear slope of flux vs. TMP, SF) of new, fouled, and cleaned RO membranes was measured via filtration test using pure water, under a temperature of 20 °C, a CFV of 1 m/s, and different TMP of 0.5–2.5 MPa.
(2) Testing and Optimization of Membrane Cleaning Program. For the above-mentioned fouled RO membranes, the single forward flushing with pure water, acid solution, alkaline solution, and sodium hypochlorite with low active chlorine concentration was conducted with the details shown in Table 2. Combined forward flushing was further conducted, with the details shown in Table 3. The forward flushing was conducted via circulating cleaning solution along RO membrane surface and stopping filtration.

(3) Exploration of Optimal Backwashing Conditions. Based on previous studies [31,32] and the above-mentioned backwashing results, backwashing with improper TMP or CFV could deteriorate RO membrane rejection capability. Therefore, further optimization of backwashing parameters was conducted. Under the constant conditions of 20 °C, CFV 1 m/s, and 40 min, different backwashing TMP of 0.5, 0.25, and 0.125 MPa were firstly compared to identify the optimal TMP. Then, under the constant conditions of 20 °C, 0.125 MPa, and 40 min, different CFV of 1.5, 1.0, and 0.5 m/s were further compared to determine the optimal CFV.
(4) Demonstration of the Optimal Combined Cleaning Method. Following the above-mentioned investigation, the optimal combined acid → alkaline forward flushing with pure water backwashing was employed for the fouled RO membrane after 6 batches of filtration to demonstrate its cleaning performance.

## 3. Results and Discussion

### 3.1. RO Membrane Fouling Potential for TDFW Reuse Application

**Figure 2:**
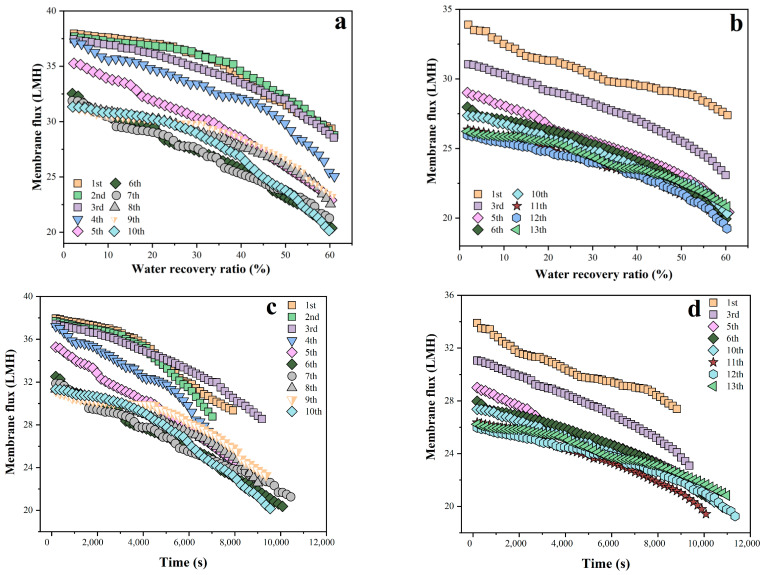
Variation in RO flux with water recovery ratio (**a**,**b**) and filtration time (**c**,**d**) during the 10/13 batches of filtration.

A 10-batch filtration was firstly conducted with the results shown in Figure 2a,c. The whole membrane flux showed no significant change in Batches 1–3, consistent with previous repeated filtration results [25]. The membrane flux slightly decreased in Batch 4, and continued to decline in Batches 5 and 6. Batches 7–10 had no significant variation in membrane flux, even slightly exceeding that of Batch 6. From Table 4, the average RO flux of batch 6 was 23.17% lower than that of Batch 1, indicating a significant permeability decrease caused by membrane fouling development. Based on the manufacturer’s specifications and test data from previous studies [25,33], when the membrane permeate production reached 625 L/m^2^ (i.e., after 5 batches), the accumulated fouling could lead to a permeability decline of more than 15%. To verify this value, another 13-batch filtration was repeated with results as shown in Figure 2b,d, where only partial results were presented to clearly illustrate the membrane flux variation. Similar to the 10-batch filtration, the membrane flux decreased significantly after 5 batches, showed no significant variation in Batches 6–10, and further slightly decreased in Batches 11–13. From Table 4, the average RO flux of Batch 6 was 19.06% lower than that of Batch 1 during the 13-batch filtration, thus confirming that RO production of 625 L/m^2^ could be regarded as the critical value causing significant fouling development and requiring membrane cleaning. It should be pointed out that the judgment on RO permeate production of 625 L/m^2^ as a critical limit for significant membrane fouling development and the resultant cleaning in this study is a specific case that is highly dependent on the RO membrane, feed quality, and operational parameters.

### 3.2. Testing and Optimization of Fouled RO Membrane Cleaning Program

**Figure 3:**
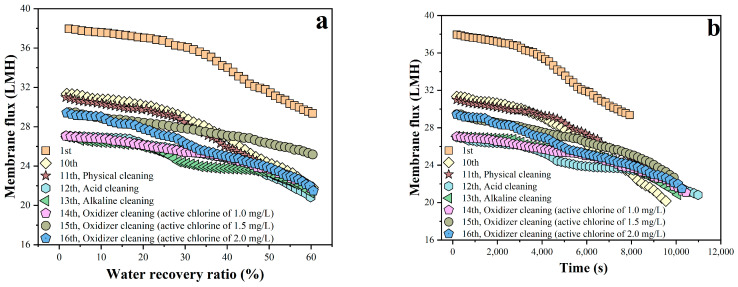
Variation in RO flux with water recovery ratio (**a**) and filtration time (**b**) under different single cleaning methods.

Membrane cleaning tests were performed on fouled membranes from Batch 10 during the 10-batch filtration. The cleaning was conducted via forward flushing, which consisted of pure water, acid, alkaline, and sodium hypochlorite solutions with a low active chlorine concentration of 1.0, 1.5, and 2.0 mg/L in sequence. The results are presented in Figure 3 and Table 5. After pure water flushing (i.e., physical cleaning), Batch 11 exhibited a slightly higher average membrane flux of 4.34% compared to Batch 10, indicating its limited cleaning performance. Following acid flushing, Batch 12 showed a pronounced decrease in flux relative to Batch 11, likely due to the accumulation of membrane fouling beyond a critical threshold, which accelerated flux decrease. In Batch 13, after alkaline flushing, only a slightly higher flux of 3.08% was observed compared to Batch 12, also showing its limited cleaning performance. When oxidative cleaning was applied with 1.0 mg/L active chlorine in Batch 14, the membrane flux remained nearly constant compared to Batch 13. Increasing the active chlorine concentration to 1.5 mg/L in Batch 15 led to a minor increase in flux of 7.51% compared to Batch 14, but with higher permeate conductivity of 76.5 μS/cm. However, 2.0 mg/L active chlorine in Batch 16 resulted in a slightly lower flux of 3.18% compared to Batch 15, but came along with a significant rise in permeate conductivity to 619 μS/cm. These findings suggested that the higher active chlorine concentration could damage this RO membrane structure, thus deteriorating its ion rejection capacity and resulting in elevated permeate conductivity, which were in agreement with the well-known fact that the dominant polyamide-based RO membrane is sensitive to deterioration by chlorine attack [34,35,36]. On the whole, the cleaning performance of the above-mentioned single cleaning methods was very limited, where the RO membrane flux after cleaning was only recovered up to that of Batch 10 and far below that of Batch 1 with a new RO membrane, indicating the complexity of RO membrane fouling and difficulty of cleaning [30,37,38].

**Figure 4:**
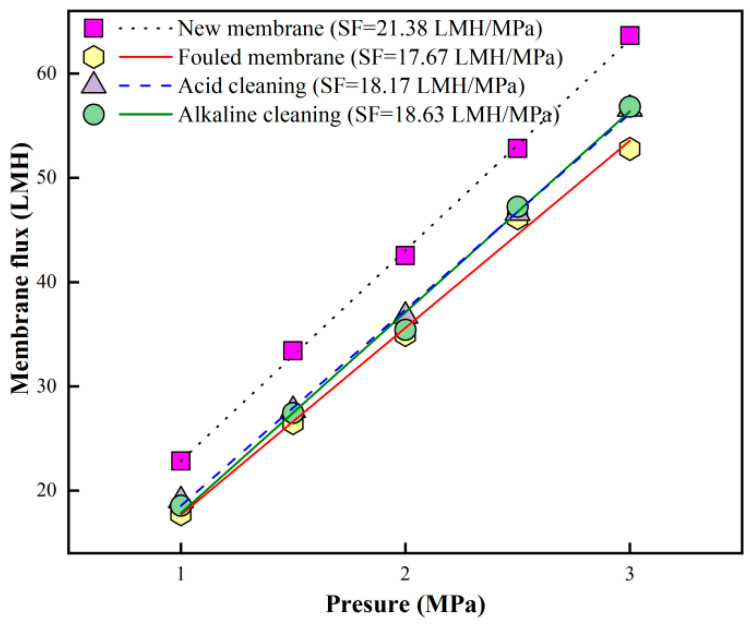
Pure water flux at different TMP of new, fouled, and cleaned RO membranes via combined acid → alkaline forward flushing.

Based on the very limited cleaning performance of the above-mentioned single forward flushing methods, combined acid → alkaline flushing (Plan 1) was employed for the fouled RO membrane after six batches of filtration in order to improve cleaning performance based on the promising cleaning performance in previous studies [29,39]. The results are summarized in Figure 4. Analysis of the membrane flux variation at different TMP revealed that the specific flux (SF) of the new membrane decreased from an initial value of 21.38 LMH/MPa to 17.67 LMH/MPa after six batches of filtration, retaining only 82.5% of its original permeability. Acid cleaning restored the SF to 18.17 LMH/MPa, corresponding to 85% of the initial SF, while subsequent alkaline cleaning further increased it to 18.63 LMH/MPa, recovering 87.2% of the new membrane’s SF. These results suggest that the combined acid → alkaline flushing strategy exhibited limited effectiveness in restoring RO membrane permeability under the tested conditions in this study, which might be due to the specific combination of the feed and RO membrane.

**Figure 5:**
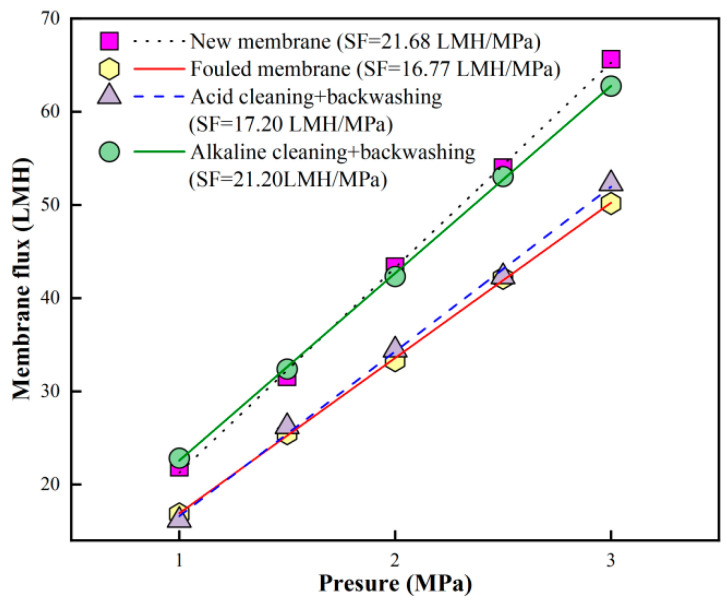
Pure water flux at different TMP of new, fouled, and cleaned RO membranes via combined acid → alkaline forward flushing and pure water backwashing.

A combined acid → alkaline forward flushing and pure water backwashing protocol (Plan 2) was employed for the fouled RO membrane after six batches of filtration in order to further improve cleaning performance based on the promising backwash performance in previous studies [40,41]. The results are presented in Figure 5. The initial SF of the new membrane was 21.68 LMH/MPa, which declined to 16.77 LMH/MPa after six filtration batches, corresponding to approximately 77.4% of the original SF. After acid forward flushing and pure water backwashing, the SF recovered to 17.20 LMH/MPa, restoring it to 79.3% of the initial SF. Subsequent alkaline forward flushing and pure water backwashing further enhanced the SF recovery to 21.20 LMH/MPa, reaching 97.8% of the new membrane’s SF. These results demonstrated that the modified cleaning strategy (i.e., combined acid → alkaline forward flushing and pure water backwashing) was highly effective and led to significant permeability restoration of fouled RO membranes in this study.

### 3.3. Exploration of Optimal Backwashing Conditions

**Figure 6:**
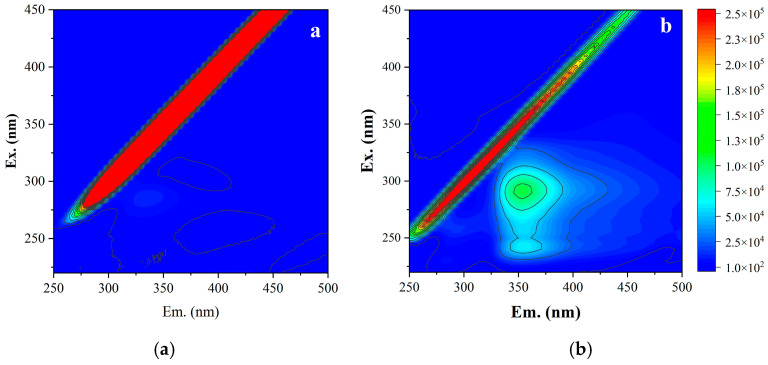
Fluorescence spectra of backwashing effluent after acid cleaning (**a**) and alkaline cleaning (**b**).

Compared to the backwashing effluent after acid cleaning (Figure 6a), the backwashing effluent after alkaline cleaning (Figure 6b) contained significantly higher levels of organics, primarily consisting of protein-like substances and soluble microbial products (SMPs). Combined with the aforementioned cleaning test results, this further confirmed that organic foulants on the RO membrane surface were the primary cause of reduced membrane permeability.

**Figure 7:**
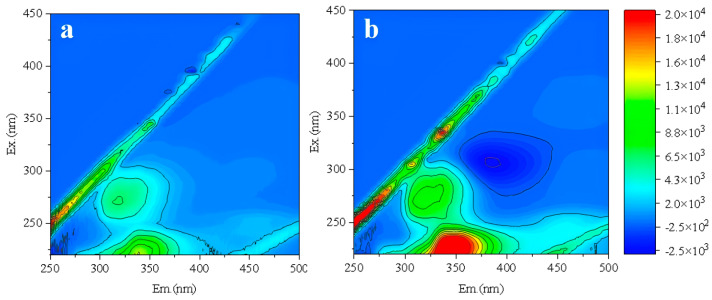
Fluorescence spectra of RO membrane effluent from the 6th batch (**a**) and after backwashing at TMP 0.5 MPa and CFV 1.5 m/s (**b**).

The RO membrane used in the experiment was a polyamide-based membrane, which was an asymmetric membrane with a non-robust supporting layer on the permeate side [42,43]. Considering the risk of mechanical damage to the supporting layer during backwashing, further refinement of the backwashing conditions was required. Based on 3D-FEEM analysis, compared with the filtered effluent after the 6th batch (Figure 7a), the organic matter content in the membrane effluent after backwashing increased significantly, as shown in Figure 7b. Therefore, the excessively high backwashing TMP and CFV could cause some damage to the membrane structure, resulting in a reduced rejection efficiency of organic matter by the RO membrane.

**Figure 8:**
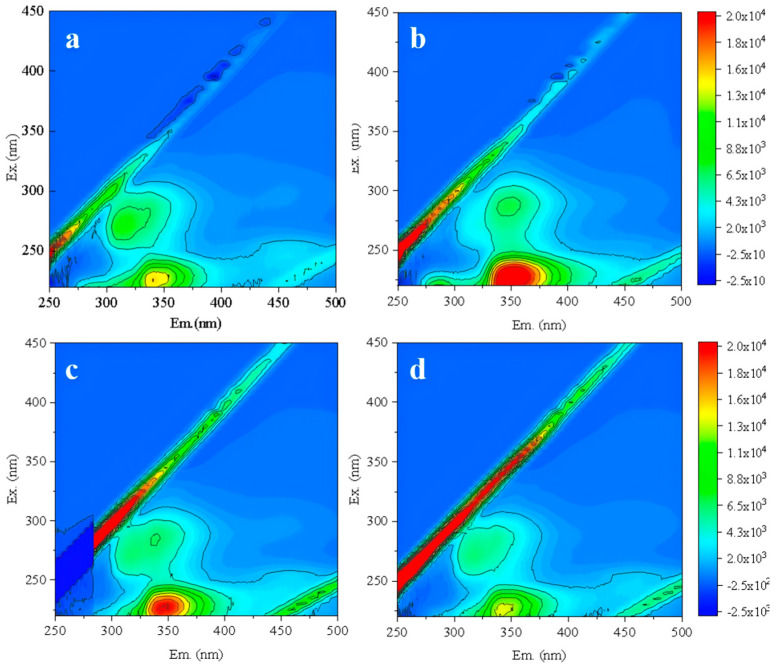
Fluorescence spectra of new RO membrane effluent without backwashing (**a**) and with backwashing TMP of 0.5 MPa (**b**), 0.25 MPa (**c**), and 0.125 MPa (**d**).

Further optimization of backwashing parameters was conducted. Under the constant conditions of 20 °C, CFV 1 m/s, and 40 min, different backwashing TMPs of 0.5, 0.25, and 0.125 MPa were firstly compared to identify the optimal TMP. The new RO membrane was backwashed under these conditions, followed by one batch filtration test. The effluent was analyzed by 3D-FEEM (Figure 8). As backwashing TMP increased, protein-like substances in the effluent gradually rose (Figure 8b,d). This indicated that higher TMP caused more damage to the RO membrane structure [44]. When the TMP was 0.125 MPa (Figure 8d), the organic content in the effluent was comparable to the new membrane without backwashing. Thus, it could be determined that the optimal backwashing TMP was 0.125 MPa.

**Figure 9:**
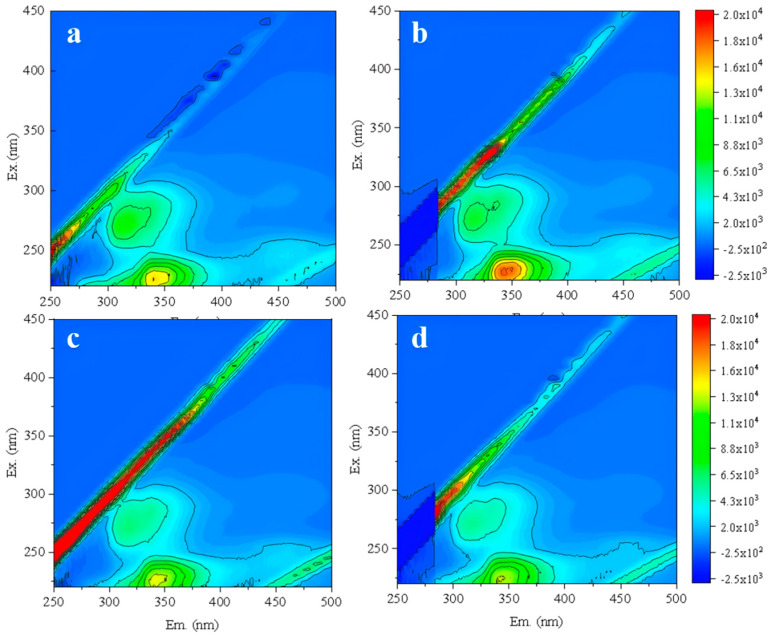
Fluorescence spectra of new RO membrane effluent without backwashing (**a**) and with backwashing CFV of 1.5 m/s (**b**), 1 m/s (**c**), and 0.5 m/s (**d**).

Then, under the constant conditions of 20 °C, 0.125 MPa and 40 min, different CFV of 1.5, 1.0, and 0.5 m/s were further compared to determine the optimal CFV. The new RO membrane was backwashed under these conditions, followed by one batch filtration test. The effluent was analyzed by 3D-FEEM (Figure 9). Compared to the filtered effluent from RO membrane without backwashing (Figure 9a), a higher CFV resulted in more membrane structural damage (Figure 9b,c) [45]. When the CFV was 0.5 m/s (Figure 9d), the organic matter content in the effluent showed no significant change compared to the new membrane. Therefore, the optimal backwashing CFV was determined to be 0.5 m/s.

### 3.4. Demonstration of the Optimal Combined Cleaning Method

**Figure 10:**
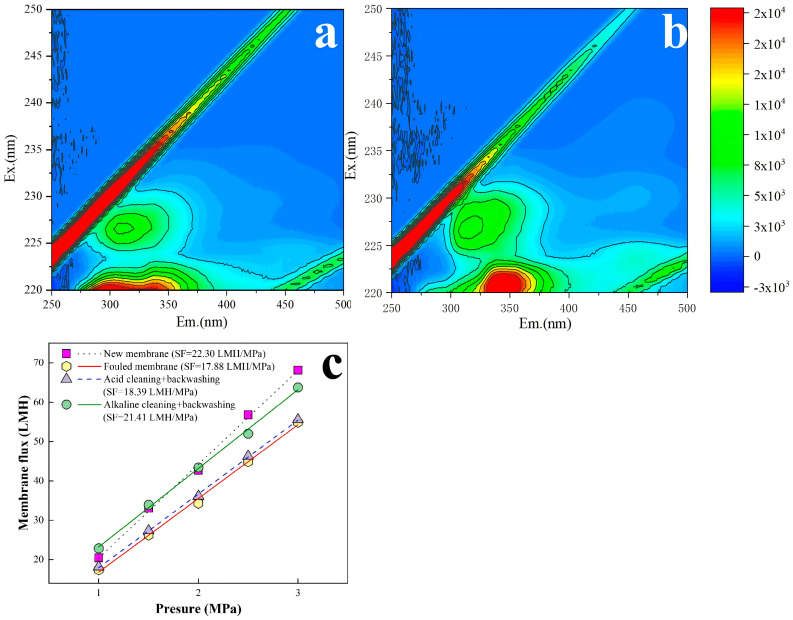
Results from the optimal combined cleaning method including fluorescence spectra of membrane permeate after 6 batches of filtration (**a**) and after combined cleaning (**b**); specific flux (**c**).

Based on the above-mentioned results, the optimal combined cleaning method (i.e., acid → alkaline forward flushing and pure water backwashing with a TMP of 0.125 MPa and CFV of 0.5 m/s) was employed for the fouled RO membrane after six batches of filtration to demonstrate its cleaning performance. The results are presented in Figure 10. From Figure 10a, the initial SF of the new membrane was 22.30 LMH/MPa, which declined to 17.88 LMH/MPa after six filtration batches, corresponding to approximately 80.2% of the original SF. After acid forward flushing and pure water backwashing, the SF recovered to 18.39 LMH/MPa, restoring it to 82.5% of the initial SF. Subsequent alkaline forward flushing and pure water backwashing further enhanced the SF recovery to 21.41 LMH/MPa, reaching 96.0% of the new membrane’s SF. Comparing Figure 10b and 10c, there were no significant changes in fluorescent organics before and after the optimal combined cleaning, indicating no damage of the RO membrane structure caused by the optimal combined cleaning. A preliminary chemical cost analysis for the optimal combined cleaning is shown in Table 6. Total chemical cost per RO production is 0.763 CNY/m^3^ with STPP, EDTA, and critic acid contributions of 42.6%, 41.9%, and 12.2%, respectively. In addition, from Figure 4, Figure 5 and Figure 10, the acid forward flushing and pure water backwashing showed limited cleaning performance and might be removed for cost reductions in both cleaning reagents and time. The alkaline forward flushing and pure water backwashing were the main contributors for cleaning performance and might be simplified as direct alkaline backwashing for cost reductions.

## 4. Conclusions

This study investigated the membrane fouling potential and cleaning performance of typical RO membranes for textile dyeing wastewater reuse applications via multi-batch filtration and cleaning experiments. Both 10-batch and 13-batch filtration tests revealed that an RO cumulative permeate volume of 625 L/m^2^ at a water recovery ratio of 60% could result in RO membrane permeability declining by more than 15%, thus meeting the general requirement for membrane cleaning in the RO industry. Protein-like substances and soluble microbial products were identified as the primary organic foulants via 3D-FEEM. The cleaning tests for the fouled RO membrane after 10 batches of filtration showed that there was very limited cleaning performance of single forward flushing with pure water, acid solution with pH of 3.5, alkaline solution with pH of 10.5, and sodium hypochlorite with low active chlorine concentrations of 1–2 mg/L, except for sodium hypochlorite cleaning with 1.5 mg/L active chlorine, which partially restored permeability. Moreover, sodium hypochlorite cleaning with active chlorine of 1.5–2 mg/L was damaging to membrane structure and thus deteriorated effluent quality. The combined acid (pH of 2) → alkaline (pH of 11.5) forward flushing method still yielded limited cleaning performance (specific flux recovered to only 85% of original levels after acid cleaning and 87.2% after alkaline cleaning). The combined acid → alkaline forward flushing and intensive pure water backwashing effectively restored specific flux up to 97.8% but deteriorated membrane rejection capability. Further optimization determined the optimal backwashing TMP of 0.125 MPa and CFV of 0.5 m/s. The optimal combined acid → alkaline forward flushing and pure water backwashing effectively restored specific flux up to 96% without deteriorating membrane rejection capabilities. The preliminary chemical cost analysis for RO membrane cleaning revealed that total chemical cost per RO production is 0.763 CNY/m^3^ with STPP, EDTA, and critic acid contributions of 42.6%, 41.9%, and 12.2%, respectively. Acid cleaning could be removed due to its very limited cleaning performance, while alkaline concentration might be reduced via chemical backwash, both further reducing chemical costs.

## Figures and Tables

**Table 1:** RO feed quality and the textile reuse standard for RO permeate in China.

Water Type	pH	COD (mg/L)	TOC (mg/L)	SS (mg/L)	Chroma (Dilution Times)	Fe (mg/L)	Mn (mg/L)	Total Hardness as CaCO_3_ (mg/L)	Conductivity (mS/cm)
RO feed	8.52	80	12	0	95	0.27	0.06	422	8.82
Reuse standard	6.5–8.5	≤50	-	≤30	≤25	≤0.3	≤0.2	≤450	≤2.5

**Table 2:** Single forward flushing for fouled RO membrane cleaning.

Cleaning Types	Physical Cleaning	Acid Cleaning	Alkaline Cleaning	Oxidizer Cleaning
Cleaning solution	Pure water	Nitric acid solution with a pH of 3.5	Commercial alkaline cleaning agents with a mass concentration of approximately 0.5%, adjusted to a pH of 10.5	Sodium hypochlorite solutions with active chlorine concentration of 1.0, 1.5, and 2.0 mg/L, respectively
Operating conditions	CFV 1.5 m/s, 20 °C, 40-min circulating cleaning	CFV 1.0 m/s, 35 °C, 40 min circulating cleaning		CFV 1.0 m/s, 30 °C, 40 min circulating cleaning

**Table 3:** Combined forward flushing and backwashing for fouled RO membrane cleaning.

Cleaning Types	Acid → Alkaline Forward Flushing (Plan 1)		Acid → Alkaline Forward Flushing with Pure Water Backwashing (Plan 2)	
Cleaning solution	1% citric acid, pH = 2, 30 ± 2 °C	2% sodium tripolyphosphate (STPP), 1% EDTA, 0.3% NaOH, 0.02% sodium dodecylbenzenesulfonate (SDBS), pH = 11.5, 35 ± 2 °C	1% citric acid, pH = 2, 30 ± 2 °C	2% STPP, 1% EDTA, 0.3% NaOH, 0.02% SDBS, pH = 11.5, 35 ± 2 °C
Operating conditions	Circulating the fouled membrane in a low-flow acid/alkaline flushing at CFV 0.75 m/s for 30/60 min with pH 2/11.5. Subsequently, halting the system and immersing the fouled membrane in an acid/alkaline solution for 1/2 h). Then, restarting the system and circulating the membrane in a high-flow pure water flushing at CFV 1.5 m/s for 0.5/1 h.		Pure water backwashing for 40 min at 0.5 MPa, 20 °C and CFV 1.5 m/s serves as the subsequent step for each cleaning phase in Plan 1.	

**Table 4:** Average RO flux of each filtration batch in Figure 2.

Batch No.	1	2	3	4	5	6	7	8	9	10	11	12	13
Average RO flux (LMH)	34.52	34.69	33.95	32.34	29.35	26.52	26.50	28.16	28.45	27.13	-	-	-
	30.38	28.30	27.71	25.97	25.03	24.59	25.89	25.90	25.72	24.35	23.60	23.43	24.02

**Table 5:** Average membrane flux in each filtration batch in Figure 3 with different single cleaning methods.

Batch No.	1	10	11	12	13	14	15	16
Cleaning method	-	-	Physical	Acid	Alkaline	Oxidizer with active chlorine of 1.0 mg/L	Oxidizer with active chlorine of 1.5 mg/L	Oxidizer with active chlorine of 2.0 mg/L
Average flux (LMH)	34.52	27.13	28.31	24.36	25.11	24.90	26.77	25.92

**Table 6:** Chemical cost analysis for RO membrane cleaning in this study.

Chemicals	Concentration (g/L)	Volume per RO Membrane Area (L/m^2^)	Price * (CNY/kg)	Production per RO Membrane Area Before Cleaning (L/m^2^)	Cost per RO Production (CNY/m^3^)	Cost Ratio (%)
Critic acid	10	2	3.5	750	0.093	12.2
STPP	20		6.1		0.325	42.6
EDTA	10		12		0.320	41.9
NaOH	3		2.1		0.017	2.2
SDBS	0.2		15		0.008	1.1
Total	-	-	-	-	0.763	100

* Price was adapted from https://www.alibaba.com/ (accessed on 8 December 2025).

## Data Availability

The original contributions presented in this study are included in the article. Further inquiries can be directed to the corresponding authors.
